# Increased surfactant protein‐D levels in the airways of preterm neonates with sepsis indicated responses to infectious challenges

**DOI:** 10.1111/apa.14630

**Published:** 2019-01-08

**Authors:** Rose‐Marie A. Mackay, J. Paul Townsend, Jennifer Calvert, Mark Anthony, Andrew R. Wilkinson, Anthony D. Postle, Howard W. Clark, David A. Todd

**Affiliations:** ^1^ Faculty of Medicine Child Health, Academic Unit of Clinical and Experimental Sciences University of Southampton Southampton UK; ^2^ Respiratory Biomedical Research Unit Southampton NIHR Southampton UK; ^3^ Neonatal Intensive Care Unit Princess Anne Hospital University Hospital Southampton NHS Foundation Trust Southampton UK; ^4^ Neonatal Intensive Care Unit Cardiff and Vale University Health Board University Hospital of Wales Cardiff Wales UK; ^5^ Neonatal Intensive Care Unit Department of Paediatrics John Radcliffe Hospital University of Oxford Oxford UK; ^6^ Department of Neonatology Centenary Hospital Canberra ACT Australia

**Keywords:** Phosphatidylcholine, Preterm neonates, Sepsis, Surfactant protein‐D, Ventilation

## Abstract

**Aim:**

Sepsis is multifactorial and potentially devastating for preterm neonates. Changes in surfactant protein‐D (SP‐D), phosphatidylcholine (PC) and PC molecular species during infection may indicate innate immunity or inflammation during sepsis. We aimed to compare these important pulmonary molecules in ventilated neonates without or with sepsis.

**Methods:**

Endotracheal aspirates were collected from preterm neonates born at 23–35 weeks and admitted to the neonatal intensive care unit at the John Radcliffe Hospital, Oxford, UK, from October 2000 to March 2002. Samples were collected at one day to 30 days and analysed for SP‐D, total PC and PC molecular species concentrations using enzyme‐linked immunosorbent assay and mass spectrometry.

**Results:**

We found that 8/54 (14.8%) neonates developed sepsis. SP‐D (p < 0.0001), mono‐ and di‐unsaturated PC were significantly increased (p = 0.05), and polyunsaturated PC was significantly decreased (p < 0.01) during sepsis compared to controls. SP‐D:PC ratios were significantly increased during sepsis (p < 0.001), and SP‐D concentrations were directly related to gestational age in neonates with sepsis (r^2^ = 0.389, p < 0.01).

**Conclusion:**

Increased SP‐D levels and changes in PC molecular species during sepsis were consistent with direct or indirect pulmonary inflammatory processes. Very preterm neonates we able to mount an acute inflammatory innate immune response to infectious challenges, despite low levels of surfactant proteins at birth.

**Abbreviations**
DPPC: Dipalmitoylphosphatidylcholine
ELISA: Enzyme‐linked immunosorbent assay
EOS: Early‐onset sepsis
ETA: Endotracheal aspirate
GA: Gestational age
GBS: Group B streptococcus
LOS: Late‐onset sepsis
PC: Phosphatidylcholine
rfhSP‐D: Recombinant fragment human SP‐D
SP‐D: Surfactant protein‐D

**Key notes**

- Sepsis can be devastating in preterm infants, and this study examined important pulmonary molecules that could provide markers of innate immunity or inflammation in this multifactorial disease.
- Endotracheal aspirates were collected from 54 preterm neonates born at 23–35 weeks and admitted to a UK neonatal intensive care unit.
- Surfactant protein‐D and phospholipids were significantly altered during sepsis and may help to identify the mechanisms involved and inform future treatment strategies.

## Introduction

Sepsis is often devastating in neonates, and many survivors develop long‐term complications, with poor neurodevelopmental outcomes 1. Studies have shown an inverse correlation between sepsis, gestational age (GA) and birthweight 2. Clinical diagnoses of sepsis are based on nonspecific symptoms, combined with specific clinical and laboratory factors that indicate infection and inflammation 3. Low levels of innate immune opsonins, such as SP‐D, are seen in preterm neonates and may contribute to increased susceptibility to infection 4. SP‐D is rapidly synthesised in the lung by alveolar cells in response to infectious challenge where it provides both pro‐inflammatory and anti‐inflammatory regulation 5, 6. One study showed that SP‐D concentrations were increased in the bronchoalveolar lavage of preterm infants ventilated for respiratory distress syndrome who received natural or synthetic surfactants during the first week of life. However, neonates who were still on supplemental oxygen on day 28 demonstrated significantly decreased levels on days two and three of life 7. We have previously shown that neonates who developed chronic lung disease demonstrated a significant reduction in SP‐D levels on day one of life, which was related to the oligomeric state of the molecule 8. Another study, of children with acute lung injuries, showed that SP‐D and SP‐D degradation products were increased in plasma and bronchoalveolar lavage 9.

Premature neonates are surfactant deficient and require surfactant administration, but current surfactant therapies do not contain SP‐D 10, 11. At present, there are little data on surfactant phosphatidylcholine (PC) during sepsis. PC is major component of biological membranes and plays a role in membrane‐mediated cell signalling, such as neurotransmission and PC transfer. Protein activation of other enzymes is also a major component of pulmonary surfactant and is often used in the lecithin‐to‐sphingomyelin ratio to calculate foetal lung maturity. Neonates with respiratory distress syndrome had lower dipalmitoylphosphatidylcholine [(DPPC) which is the major surface tension lowering PC], than those without 10, and 24 hours after natural or synthetic surfactant administration, there was no significant difference in surfactant PC composition in babies with respiratory distress syndrome, including DPPC 11. However, in children with acute lung injuries, the response to sepsis included significant reductions in total PC and surfactant PC species, including DPPC. These children also experienced a rise in mono‐ and di‐unsaturated PC, which was probably associated with altered surface membrane properties, including surface tension 9.

The aim of this study was to compare alterations in SP‐D, PC and PC molecular species in preterm neonates during infection by studying the endotracheal aspirates (ETAs) from ventilated neonates without or with sepsis. We speculated that that there would be no difference between early‐onset sepsis (EOS) and late‐onset sepsis (LOS).

## Materials and methods

### Study cohort

Endotracheal aspirates were obtained from all mechanically ventilated premature neonates who were born at <36 weeks of GA and admitted to the Neonatal Intensive Care Unit at the John Radcliffe Hospital, Oxford, UK, from 9 October 2000 to 22 March 2002. All neonates were included unless they had a congenital abnormality. The final sample comprised of 54 premature neonates born at 25–35 weeks of GA. There were 46 without sepsis and eight with sepsis with a mean GA of 29.6 ± 3.2 and 28.1 ± 3.1, respectively (Table 1).

**Table 1:** Baseline characteristics of the 54 neonates included in the study without (46) and with (8) sepsis

	No sepsis n = 46*	Sepsis n = 8	p Value
Gestational age	29.6 ± 3.2	28.1 ± 3.1	ns
Male:Female ratio	29:17	4:4	ns
Age at sample collection (days)	1–30	1–20	ns
Number of endotracheal aspirate (ETA) samples	91	21	
Surfactant PC (%)§	71.7 (61.9–81.0)†	69.8 (57.6–77.4)	
	OR 0.964, 95% CI 0.909–1.023‡		ns
Mono‐/Di‐unsaturated PC (%)¶	18.1 (14.6–21.8)	19.6 (16.7–25.3)	
	OR 1.096, 95% CI 1.00–1.203		0.05
Polyunsaturated PC (%)**	4.8 (2.7–7.1)	2.4 (1.3–3.5)	
	OR 0.721, 95% CI 0.565–0.920		<0.01

GA, Gestational age; M:F, Male: female.

Analysis studied: (i) surfactant PC, (ii) mono‐unsaturated and di‐unsaturated PC, (iii) polyunsaturated PC. All 46 neonates with no sepsis versus eight neonates with sepsis.

* n, number of neonates.

† Median [interquartile range (IQR)].

‡ Generalised linear model adjusted for repeated sampling with neonates and GA and presented as OR, odds ratio, CI, confidence interval.

§ Surfactant PC: PC(14:0/16:0); PC(16:0A/16:1); PC(14:0/18:2); PC(16:0/16:1); PC[16:0/16:0 (DPPC)].

¶ Mono‐unsaturated/di‐unsaturated PC: PC(16:0/18:2); PC(16:0/18:1); PC(18:1/18:2); PC(18:0/18:2).

** Polyunsaturated PC: PC(16:0/20:4); PC(16:0/22:6); PC(18:1/20:4); PC(18:0/20:4).

### Sample collection and processing

Specimens were taken as part of the clinical care of the neonates, which is a standard protocol that complies with the ethical standards of the Declaration of Helsinki. Tracheal tube suctioning was only performed if the infant required it and was not provided on a regular basis. We instilled 250 μL of 0.9% saline into the tracheal tube and aspirated into a collecting trap using a suction catheter, applying a suction pressure of 100 mmHg. The suction catheter was flushed with approximately 0.5 mL 0.9% saline and the sample volume made up to 1 mL. Endotracheal aspirate (ETA) samples were numbered and therefore anonymised at this stage. The ETAs were centrifuged at 400 *g* and 4°C for 10 minutes to remove the cells and cell‐free supernatant and then stored at –80°C prior to analysis.

### Surfactant protein‐D (SP‐D) ELISA

Native human SP‐D and recombinant fragment of human SP‐D (rfhSP‐D) were captured using 1:1000 dilution rabbit antirecombinant SP‐D (rb‐αrfhSP‐D) and were detected using rabbit polyclonal biotinylated α‐rfhSP‐D antibody (1:1000 dilution) by incubating at 37°C for one hour. After washing, 100 μL Streptavidin‐HRP (1:10,000) Sigma (Sigma‐Aldrich Company Ltd, Dorset, UK) was added to each well, and plates were incubated at 37°C for 30 minutes prior to washing. Finally, tetramethylbenzene substrate (Bio‐Rad laboratories Ltd, Hertfordshire, UK) was added to each well, and after sufficient colour development, the reaction was stopped with 50 μL 0.5 mol/L sulphuric acid. Absorbance values were measured at 450 nm, and background control values were subtracted from the binding protein‐coated wells, as described previously 12, 13. Samples were diluted one in 10, 20, 40, 80 or 120, adding 100 μL/well and calculated to ng/mL from a calibration standard curve using rfhSP‐D.

### Electrospray ionisation mass spectrometry

**Figure 1:**
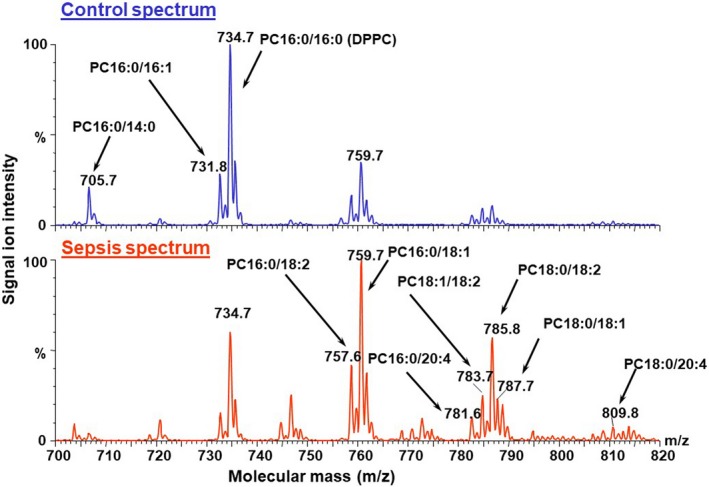
Phosphatidylcholine (PC) spectrum in neonates with: (A) no sepsis and (B) sepsis. Note the increase in the mono‐unsaturated and di‐unsaturated PC species: PC (16:0/18:2); PC (16:0/18:1); PC (18:1/18:2); PC (18:0/18:2). Note also the reduction in the polyunsaturated PC species: PC (16:0/20:4); PC (16:0/22:6); PC (18:1/20:4); PC (18:0/20:4).

Endotracheal aspirates were processed for phospholipid analysis with electrospray ionisation mass spectrometry by lipid extraction using a modified Bligh and Dyer method as described previously 9, 14. Lipids were extracted from the remaining ~950 μL ETAs by a chloroform–methanol mixture following the addition of 5 nmol of dimyristoyl PC (PC14:0/14:0) as internal standard 9. Lipid extracts (chloroform–methanol) were then evaporated to dryness under nitrogen prior to mass spectrometry. Individual molecular species of PC were analysed by using a Quattro Ultima triple‐quadrupole mass spectrometer (Micromass UK Ltd, Manchester, UK). Lipid extracts were redissolved in 20 μL of chloroform–methanol (1:2 vol/vol), and 5 μL was introduced by rheodyne valve injection into a flow of methanol–chloroform–water (80:10:10 vol/vol/vol) pumped at 10 μL/min into the capillary inlet of the mass spectrometer. Total PC concentration was calculated from the sum of the individual species and used for quality control to ensure the validity of the samples. Analysis of the PC compositions at very low concentrations typically detected only the major PC species (PC16:0/16:0) and, consequently, quantification of the other PC species was not feasible in these samples. For this reason, samples were omitted from further analysis if the PC concentration was <1.5 nmol/mL, indicating an invalid sample. The mass spectrometer data were processed using the MassLynx 4.0 (Micromass UK Ltd, Manchester, UK) and Excel (Microsoft Corp, Washington, USA). Typical PC spectrums and molecular species from a neonate without and with sepsis are shown in Figure 1.

### Clinical diagnosis of sepsis

Sepsis was diagnosed based on a number of results and indicators. It was diagnosed if neonates had a positive blood culture, a diagnosis of necrotising enterocolitis with clinical and radiological signs 15 or a prenatal clinical risk factor, such as prolonged rupture of membranes >18 hours, or group B streptococcus (GBS) colonisation. A positive diagnosis was also made if there were two or more of the post‐natal clinical signs for sepsis. These included the following: temperature instability, increased oxygen requirements, increased apnoea, poor peripheral perfusion, hypotension, feed intolerance, abdominal distension, haematological changes such as a raised white cell count and or decreased platelet count and, raised C‐reactive protein that resulted in the initiation of antibiotics for sepsis. We also divided the neonates based on the diagnosis of EOS within the first three days of life or LOS if it occurred after day three of life 3, 16. There were no neonates in the four‐ to six‐day period with LOS.

### Statistics

Generalised linear models using logistic regression were used to analyse the data, and corrections were made for repeated sampling within neonates and the biased distribution of the infants with sepsis having a lower GA. We compared the SP‐D and PC concentrations between those without and with sepsis and subgrouped into EOS and LOS. Specific PC molecular species were analysed separately in three groups; surfactant PC, associated with alveolar stability PC(14:0/16:0); PC(16:0a/16:1);PC(14:0/18:2); PC(16:0/16:1); PC[16:0/16:0 (DPPC)] (Fig. 1), mono‐ and di‐unsaturated PC such as associated with plasma PC and cell membrane PC PC(16:0/18:2); PC(16:0/18:1); PC(18:1/18:2); PC(18:0/18:2) (Fig. 1), and polyunsaturated PC, for example, associated with prostaglandin production: PC(16:0/20:4); PC(16:0/22:6); PC(18:1/20:4); PC(18:0/20:4) (Fig. 1). Fisher's exact test and *t*‐test were used for basic analysis. Results were considered significant if p < 0.05 (SPSS version 20, IBM Corp., Armonk, NY, USA).

### Ethical approval

The Central Oxford Research Ethics Committee (COREC) of the John Radcliffe Hospital approved this study (CJD/dms/C99.236). Informed consent was not deemed to be necessary from the parents as the ETA specimens were to be discarded and they were anonymised at the time of collection.

## Results

We recruited 54 ventilated preterm neonates [33/54 (61%) males] born at 23‐ to 35‐week GA of which 112/150 (74.7%) ETA samples we obtained and valid for analysis. These 112 samples were obtained from one day to 30 day of life. There were no significant differences in the basic characteristics between the 46 neonates without sepsis and the eight neonates with sepsis (Table 1). In addition, there were no differences when the neonates were divided into the 40 neonates without EOS and the four neonates with EOS or the six neonates without LOS and the four neonates with LOS (Table 2). The four neonates diagnosed with EOS comprised: one with positive *E. coli* blood cultures, two with maternal GBS colonisation, but negative neonatal blood cultures, and one with prolonged rupture of membranes and green liquor. Of the four neonates diagnosed with LOS, there were as follows: three with necrotising enterocolitis and one with long line sepsis. The diagnoses at the time of ventilation included respiratory distress in the majority of the neonates in both groups, inadequate spontaneous respiratory effort, poor condition at birth, meconium aspiration, persistent pulmonary hypertension, congenital pneumonia, oesophageal atresia, necrotising enterocolitis and the development of chronic lung disease.

**Table 2:** Baseline characteristics and analysis of endotracheal aspirates (ETA) SP‐D, total surfactant PC and SP‐D:PC ratio for neonates without and with (i) early‐onset sepsis (EOS) and (ii) late‐onset sepsis (LOS)

	No EOS n = 40*	EOS n = 4	p Value	No LOS n = 6	LOS n = 4	p Value
GA	29.8 ± 2.7	30.1 ± 2.1	ns	27.8 ± 4.0	26.1 ± 2.3	ns
M:F ratio	27:13	3:1	ns	2:4	1:3	ns
Age at sample collection (days)	1–6	1–4	ns	7–30	7–20	ns
Number of endotracheal aspirate (ETA) samples	77	7		14	14	
SP‐D (ng/mL)	19.1 (4.1–145.8)†	803.0 (148.8–3150.0)		13.6 (0.3–53.3)	256.0 (63.5–436.6)	
	OR = 1.002, 95% CI 1.001–1.003‡		0.005	OR = 1.011, 95% CI 1.001–1.021		0.04
Total PC (nmol/mL)	176.7 (75.4–351.7)	127.6 (44.5–447.5)		91.0 (30.0–309.1)	63.8 (43.6–112.6)	
	Not in equation		ns	Not in equation		ns
SP‐D:PC ratio	0.16 (0.03–0.73)	5.6 (0.20–8.55)		0.29 (0.0–1.4)	3.5 (2.50–6.35)	
	OR 1.848, 95% CI 1.240–2.165		0.003	OR 1.872, 95% CI 1.117–3.136		0.02

GA, Gestational age; M:F, Male:female; OR, Odds ratio; CI, Confidence interval.

Early‐onset sepsis (EOS): 40 neonates with no EOS versus four neonates with EOS (less than four days at the onset of sepsis). Late‐onset sepsis **(**LOS): six neonates with no LOS versus four neonates with LOS (greater than three days at the onset of sepsis).

* n = number of neonates.

† Median [interquartile range (IQR)].

‡ Generalised linear model adjusted for repeated sampling within neonates and for GA and gender.

**Figure 2:**
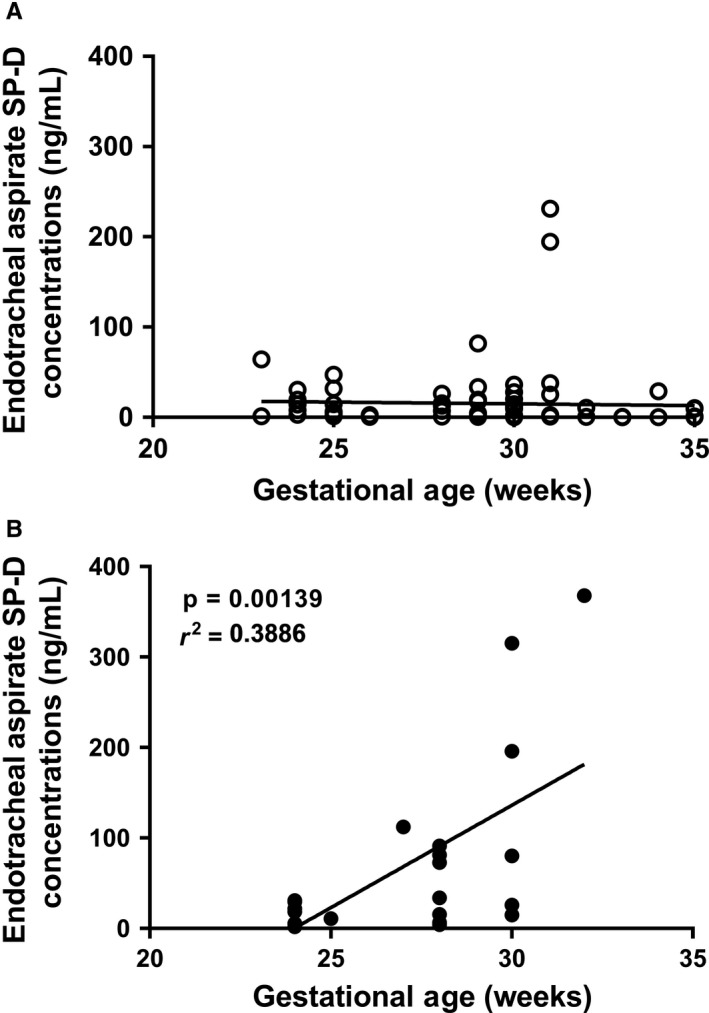
Graph of SP‐D level against gestation in neonates with: (A) no sepsis and (B) with sepsis. Note the linear increase with increasing GA in neonates with sepsis (B).

There was no correlation between SP‐D levels and GA, for those neonates without sepsis (Fig. 2A). However, there was a significant increase in SP‐D concentration in the eight neonates with sepsis with a significant correlation to GA, median (IQR) SP‐D; 19.1 ng/mL (4.1–134.5) without sepsis versus 294.1 ng/mL, (148–80) with sepsis, 1.004 odds ratio [OR], 1.001–1.006 95% confidence interval (95% CI), p < 0.001, r^2^ = 0.389, p < 0.01 (Fig. 2B).

There was no association between PC levels and GA. Similarly, there was no significant change in total PC concentrations in the 46 neonates without sepsis compared to the eight neonates with sepsis [median total PC: 170.7 nmol/mL 64.8–312.9 (IQR) versus 71.2 nmol/mL, 50.8–127.6 (IQR), 0.999 (OR), 0.996–1.001(95% CI), respectively, nonsignificant]. This was regardless of whether there was diagnosis of EOS or LOS (Table 2). However, the SP‐D/PC ratio, corrected for overall surfactant recovery in the ETAs, was significantly increased in neonates with sepsis [median SP‐D/PC ratio: 0.2, 0.0–0.8 (IQR) without sepsis versus 3.6, 2.3–7.0 (IQR) with sepsis, 1.89 (OR), 1.374–2.599 (95% CI), p < 0.0001]. These changes in the SP‐D/PC ratio occurred regardless of whether the sepsis was EOS or LOS (Table 2).

Although there was no significant difference in total and surfactant PC in neonates during sepsis (Tables 1 and 2), there were significantly increased changes in mono‐unsaturated and di‐unsaturated PC and significantly lower polyunsaturated PC during sepsis (Fig. 1, Table 1).

## Discussion

We have shown for the first time that preterm neonates with sepsis demonstrate a significant increase in pulmonary SP‐D with no significant change in total PC. There was no significant difference in saturated PC in neonates with sepsis (Table 1). Meanwhile, mono‐unsaturated and di‐unsaturated PC, which are associated with plasma PC and white blood cell membranes and thus the inflammatory process (Table 1, Fig. 1), were significantly higher and polyunsaturated PC, associated with prostaglandin synthesis, significantly lower during sepsis (Table 1, Fig. 1). The changes in the pulmonary inflammatory process, with a rise in SP‐D and change in PC molecular species, were consistent with a study of children who developed an acute lung injury 9. Our study shows, for the first time, that premature neonates with sepsis are capable of mounting an innate immune inflammatory response that was more pronounced with increased GA, but not related to GA *per se,* as shown in the babies without sepsis (Fig. 2). In neonates with infection, the significant correlation with GA suggested that the more mature infants (30‐ to 35‐week GA) were able to mount a greater SP‐D response than the less mature infants (<30 weeks’ GA) and this increase in SP‐D was present in both the neonates with EOS and LOS (Table 2).

The SP‐D increase during sepsis supports the view that SP‐D responds to infection as an acute phase reactant. Two studies have shown that premature neonates who showed a significant reduction in SP‐D levels on day one of life 8 and days two and three of life 7 went on to develop chronic lung disease 7. Sepsis is known to be associated with chronic lung disease 17, but further work is needed to determine whether decreased pulmonary SP‐D levels are associated with the development of chronic lung disease or simply a response to the modulating inflammatory process of sepsis. This response, in turn, may lead to changes to the pulmonary epithelium, with cellular infiltration leading to chronic lung disease 18, 19. When pulmonary epithelium is damaged increased SP‐D is detected in blood, this may provide a more rapid diagnosis and allow targeted drug regimens that could prevent disease progression more effectively 20. Studies have shown that increased SP‐D levels can be detected in the bronchoalveolar lavage of children who develop ventilator‐associated pneumonia, before there is any pathological or culture‐based evidence of pulmonary infection 21. SP‐D interacts with common pathogens, associated with pneumonia, such as *Pseudomonas aeroginosa* by agglutinating and trapping to promote the removal of the bacteria from the pulmonary compartment 22. SP‐D also opsonises *Klebsiella pneumonia* to enhance its removal and agglutinates *Staphylococcus aureus‐*enhancing neutrophil uptake 23. However, these interactions are accompanied by protease SP‐D degradation from host immune cells or pathogen virulent factors, thus reducing SP‐D mediated defence but allowing potentially cleaved fragments of SP‐D to enter the blood 8. We have previously reported that serum SP‐D levels and SP‐D breakdown products correlated with lung epithelial damage in children with acute lung injury and sepsis 9.

There are limitations in current detection and monitoring methods for sepsis. For example, increases in C‐reactive protein, total white blood cell counts and differential white blood cell counts are sensitive indicators of general inflammation, and not sepsis, and can be elevated in noninfectious conditions, such as prolonged rupture of membranes or asphyxia 24. Therefore, there seems to be a need for a more lung‐specific biomarker of inflammation and SP‐D may provide this, given increased systemic concentrations of SP‐D are associated with more severe lung disease 13. This highlights the need for more accurate rapid methods to identify sepsis so that treatment can be provided more quickly and lead to improved long‐term outcomes. Unlike C‐reactive protein levels in serum 24, 25, the SP‐D in ETAs in our study did not rise in relation to GA when there was no evidence of infection (Fig. 2A). The levels only rose in response to infection when the concentration was significantly related to GA (Fig. 2B). This rise could possibly be used to monitor treatment responses and indeed treatment progression.

Total PC pools did not significantly change with sepsis, but there were alterations in PC molecular species. Although surfactant PC species did not change during sepsis, there were increases in mono‐unsaturated and di‐unsaturated PC species that were consistent with previous studies and decreases in polyunsaturated PC species that were inconsistent 9. Mechanisms for the increase in mono‐unsaturated and di‐unsaturated PC species have been proposed and are from at least two sources 9. First, during ventilation damage to the alveolar–capillary junction may cause capillary leak and studies have shown that plasma PC including PC(16:0/18:2) and PC(18:1/18:2) increased in the bronchoalveolar lavage of patients with sepsis 9. Second, an influx of neutrophils during acute lung injury and chronic lung disease, and release of membrane PCs, such as PC(16:0/18:1) and PC(18:0/18:1) due to damage and breakdown, may increase their concentrations 9, 17. Mechanisms for the decrease in polyunsaturated PC during sepsis are speculative. During synthesis, polyunsaturated fatty acids have been shown to be substrates of eicosanoids via the arachidonic and eicosapentaenoic acids that form part of the inflammatory process 26. Assuming that polyunsaturated PCs are consumed in this metabolic pathway in neonates with sepsis, the low polyunsaturated PC found in our study may be accounted for by this mechanism.

The strengths of our study were that the ETAs were taken from all ventilated preterm neonates on the neonatal unit unless they had a congenital abnormality and this was a prospective study. The samples were examined for consistency by mass spectrometry and removed from analysis if inconsistent. This study provided a rare opportunity to examine clinical samples from population of very premature infants which gave a unique insight to their innate immune responses. The limitations of our study were that the ETAs were not all taken at exactly the same time for all neonates but were taken as clinically required. The nature of the sample population is such that samples are precious and very rarely collected and because of this, the sample numbers are relatively low.

## Conclusion

We have shown that premature neonates were able to mount an innate immune response by increasing lung SP‐D levels in response to infections. Despite the unaltered levels of total PC, during sepsis, there was an increase in mono‐unsaturated and di‐unsaturated PC, a decrease in polyunsaturated PC and increased SP‐D levels. Increased SP‐D levels may prove to be a more specific indication of lung infection in preterm neonates, but further studies are required to explain the mechanisms of increased SP‐D and altered PC molecular species.

## Funding

This report was based on independent research partly funded by The MRC Immunochemistry Unit. University of Oxford, South Parks Road, Oxford, OX1 3QU, UK and the Southampton National Institute for Health Research Respiratory Biomedical, Research Centre.

## Conflict of interests

HWC was the author of granted patents for treating lung diseases using surfactant protein‐D (patent numbers; WO03035679A, EP1440083A2, WO03035679A2, US20040259, WO03035679A3, US20040259201A1, JP2005522988T2). The remaining authors have no potential conflict of interests to declare.
